# Algorithm for classifying the underlying pathologies in postpartum hemorrhage using bleeding rate, fibrinogen, and fibrin/fibrinogen degradation products: a case series study

**DOI:** 10.1186/s12884-026-09762-2

**Published:** 2026-08-03

**Authors:** Katsuhiko Tada, Yasunari Miyagi, Ichiro Yasuhi, Keisuke Tsumura, Ikuko Emoto, Maiko Sagawa, Norifumi Tanaka, Kyohei Yamaguchi, Kazuhisa Maeda, Kosuke Kawakami

**Affiliations:** 1 Department of Obstetrics and Gynecology, National Hospital Organization, Okayama Medical Center, 1711-1 Tamasu, Kita Ward, Okayama, 701-1192 Japan; 2 Medical Data Labo, 289-48 Yamasaki, Naka Ward, Okayama, 703-8267 Japan; 3 Department of Obstetrics and Gynecology, National Hospital Organization, Nagasaki Medical Center, 2-1001-1 Kubara, Omura, 856-0835 Japan; 4 Department of Obstetrics and Gynecology, National Hospital Organization, Saga Hospital, 1-20-1 Hinode, Saga, 849-8577 Japan; 5 Department of Obstetrics and Gynecology, National Hospital Organization, Kyoto Medical Center, 1-1 Fukasakumukaihata, Kyoto, 612-8555 Japan; 6 Takabatake Women’s Clinic, 4-18 Ogimachi, Daito, 574-0033 Japan; 7 Department of Obstetrics and Gynecology, National Hospital Organization, Kure Medical Center, 3-1 Aoyama, Kure, 737-0023 Japan; 8 Department of Obstetrics and Gynecology, National Hospital Organization, Higashihiroshima Medical Center, 513 Saijochojike, Higashihiroshima, 739-0041 Japan; 9 Division of Obstetrics and Perinatal Care for Central Hiroshima, Hiroshima University Hospital, 1-2-3 Kasumi, Minami Ward, Hiroshima, 734-8551 Japan; 10 Department of Obstetrics and Gynecology, National Hospital Organization, Mie Chuo Medical Center, 2158-5 Hisaimyojincho, Tsu, 514-1101 Japan; 11 Department of Obstetrics and Gynecology, National Hospital Organization, Shikoku Medical Center for Children and Adults, 2-1-1 Senyucho, Zentsuji, 765-8507 Japan; 12 Department of Obstetrics and Gynecology, National Hospital Organization, Kokura Medical Center, 10-1 Harugaoka, Kokuraminami Ward, Kitakyushu, 802-8533 Japan

**Keywords:** Bleeding rate, Coagulopathy, Disseminated intravascular coagulation, Fibrin/fibrinogen degradation products, Fibrinogen, Postpartum hemorrhage, Underlying pathology

## Abstract

**Background:**

Understanding patients’ underlying pathologies is essential for appropriate treatment during postpartum hemorrhage (PPH) management. The objective of this study is to develop an algorithm that quantitatively classifies the underlying pathologies in PPH using clinical parameters.

**Methods:**

A multicenter case series study involving nine perinatal centers in Japan was conducted on patients with severe PPH (blood loss > 2000 mL) excluding placental abruption, and placental abruption irrespective of blood loss spanning August 2020 to June 2024. The primary outcome was the development of an algorithm for classifying the underlying pathologies in PPH, based on bleeding rate, fibrinogen, and fibrin/fibrinogen degradation products (FDP). We used collected data to develop an algorithm by which cases with abnormally high bleeding rate (≥ 61 mL/min) were classified as whole blood loss with high bleeding rate (WBLH). Of cases with bleeding rate < 61 mL/min, those with fibrinogen ≥ 237 mg/dL were classified as non-coagulopathy (NCP). Of cases with fibrinogen < 237 mg/dL, those with FDP < 2 mg/dL and FDP ≥ 2 mg/dL were classified as whole blood loss with low bleeding rate (WBLL) and coagulation factor consumption (CFC), respectively. In CFC cases, a predictive formula distinguished between disseminated intravascular coagulation (DIC) from locally initiated and progressive consumptive coagulopathy for hemostasis (LIPC). We used the Mann–Whitney test and Fisher’s exact test for the statistical analysis.

**Results:**

Among 171 participants, comprising 131 severe PPH cases and 40 placental abruption cases, the algorithm classified cases into WBLH (*n* = 9), NCP (*n* = 88), WBLL (*n* = 12), and CFC (*n* = 62). Of the CFC cases, 3 had DIC and 59 had LIPC. FDP levels did not differ between WBLH (median, 1.19 mg/dL) and WBLL (median, 0.91 mg/dL). Hemoglobin (median, 6.3 g/dL) in WBLL and fibrinogen (median, 168.5 mg/dL) in CFC were significantly lower than for the other three categories (*P* < 0.05). Coagulation-fibrinolytic activation in DIC was significantly increased compared with LIPC cases.

**Conclusions:**

Our algorithm, using bleeding rate, fibrinogen, and FDP as clinical parameters, classified PPH cases into four underlying pathologies. This pilot study provides a foundation for pathophysiology-based clinical management of PPH.

## Introduction

Postpartum hemorrhage (PPH) remains the leading cause of maternal morbidity and mortality worldwide, even in high-income countries, causing 15% of maternal deaths [1]. Consequently, bundle care – using multiple intervention points and roles – recently has been advocated for rapid response to PPH bleeding and control [2]. Effective management requires staying mindful of patients’ underlying pathologies to provide appropriate care. Correcting coagulopathies that develop during PPH is crucial for controlling bleeding; these states are classified as loss or dilution of blood, or consumption of coagulation factors [3, 4]. The latter is further divided into two conditions. One is systemic disseminated intravascular coagulation (DIC) and another is consumptive coagulopathy localized to the uterus and/or placental bed, as addressed by Lier et al. [4] and Collis et al. [5] which has not yet been quantitatively described.

Our research group has been using specific values and a formula with fibrinogen and fibrin/fibrinogen degradation products (FDP) to investigate means of characterizing the above conditions associated with coagulopathy. In Miyagi et al. [6], based on an analysis of the biological relationship between fibrinogen and FDP using artificial intelligence, we reported the threshold criteria in severe PPH patients to initiate coagulation activation were 237 mg/dL and 2 mg/dL for fibrinogen and FDP, respectively. In Miyagi et al. [7], we used artificial intelligence to develop a boundary formula for predicting clinical DIC during delivery: FDP–fibrinogen/3–60 (mg/dL) value ≥ 0 indicates DIC; we validated the formula using cases with severe placental abruption [8]. We also have studied the significance of bleeding rate through research on PPH pathophysiology [9].

PPH management mainly applies pharmacological and surgical treatment to arrest the bleeding, along with blood products transfusions [2, 10]. A multidisciplinary approach, including non-obstetricians, is required in severe cases; therefore, all members of the care team must have a shared understanding of the patient’s underlying pathologies. Given these circumstances, in this study, we aimed to develop an algorithm for classifying the underlying pathologies associated with acute bleeding and/or coagulopathy during PPH. We used the fibrinogen and FDP threshold criteria we have reported [6, 7], and added bleeding rate. We then compared the laboratory and clinical data among underlying pathologies classified to confirm the biological plausibility of the developed algorithm.

## Methods

### Study design and participants

This multicenter case series used cases with severe PPH or placental abruption and that were managed at nine National Hospital Organization (NHO) perinatal centers in Japan from August 2020 to June 2024. The current study was approved by the NHO Central Research Ethics Committee (R1-1009002; February 7, 2020) and conducted with reference to the Strengthening the Reporting of Observational Studies in Epidemiology (STROBE) statements [11]. Written informed consent was obtained from all participants.

Because placental abruption is considered to be a typical cause of obstetrical DIC [5] that may be associated with a severe consumptive coagulopathy characterized by hypofibrinogenemia despite minimal blood loss initially [12], inclusion criteria were pregnant patients with both PPH (excluding placental abruption) of > 2000 mL blood loss within 24 h of delivery [10] and placental abruption that was clinically diagnosed irrespective of blood loss, who delivered at these centers, including those transferred to the centers before delivery. Exclusion criteria were medical complications that could cause coagulopathy and use of medications affecting the coagulation system (e.g., aspirin).

### Data collection

The attending physicians at each center obtained blood samples when deemed clinically necessary, regardless of blood loss. Hemoglobin, platelet counts, prothrombin time-international normalized ratio, fibrinogen, FDP, and D-dimer were measured in each center’s laboratory. Thrombin–antithrombin complex (TAT) and plasmin-α2–plasmin inhibitor complex (PIC) were measured at SRL Inc. (Tokyo, Japan). The working ranges for the FDP and TAT assay were ≤ 96 mg/dL and ≤ 120 ng/mL, respectively.

Blood loss amount measured by weighing of soiled gauzes and/or measuring fluid volume, time of initial blood sampling after delivery, bleeding rate, shock index, transfusion volume of blood products, administration volume of clear fluids, and cause of bleeding were the clinical data. Obstetrical management, blood products transfusion, and determination of the cause of bleeding were performed at the attending physicians’ discretion. Bleeding rate was defined as the blood loss at initial blood sampling (mL) divided by the initial blood sampling time after delivery (min). Shock index was calculated by dividing heart rate (bpm) by systolic blood pressure (mmHg).

For analysis, we used fully anonymized data from the first measurement after delivery for each patient.

### Development of an algorithm for classifying the underlying pathologies in PPH

Following data collection, we hypothesized that cases with an abnormally high bleeding rate and cases with coagulopathy (fibrinogen < 237 mg/dL) without elevated FDP (< 2 mg/dL) – criteria established in our previous study [6] – represented whole blood loss resulting from an inability to form hemostatic thrombi at the vascular injury site. Conversely, cases with coagulopathy (fibrinogen < 237 mg/dL) and elevated FDP (≥ 2 mg/dL) represented increased coagulation with subsequent consumption of coagulation factors. This coagulopathy can be further classified as DIC or local consumptive coagulopathy as non-DIC arising from different mechanisms. DIC is a rare condition characterized by systemic intravascular microthrombi formation [13] caused by not only the influx of massive tissue factors from damaged maternal desidua [14, 15] but also the involvement of the immune system [14, 16]. In contrast, most consumptive coagulopathy cases are related to the local consumption of coagulation factors to form hemostatic thrombi in the injured myometrial vessels and/or the placental bed [4, 5]. Thus, for non-DIC cases, coagulation activation might initiate and progress locally, which we defined the term *LIPC* (locally initiated and progressive consumptive coagulopathy) in this study.

To develop our algorithm, we first defined cases with bleeding rate ≥ 95th percentile of all participants as having whole blood loss with high bleeding rate (WBLH). Next, among cases with bleeding rate < 95th percentile, we defined those with fibrinogen ≥ 237 mg/dL as non-coagulopathy (NCP) and those with fibrinogen < 237 mg/dL as coagulopathy. We used an FDP threshold of 2 mg/dL to further categorize coagulopathy into two conditions: (1) FDP < 2 mg/dL had whole blood loss with low bleeding rate (WBLL) and (2) FDP ≥ 2 mg/dL had coagulation factor consumption (CFC). Finally, in severe CFC cases, we used the formula for predicting clinical DIC to diagnose DIC: a calculated value found from the formula “FDP–fibrinogen/3–60 (mg/dL)” ≥ 0 was DIC, otherwise, it was LIPC [7, 8].

### Outcomes

The development of an algorithm to classify the underlying pathologies in PPH using bleeding rate, fibrinogen, and FDP was the primary outcome. Comparing the laboratory and clinical data among each underlying pathology was the secondary endpoint.

### Data analysis

Continuous and categorical variables are expressed as median (range) and number (%), respectively. We used the Mann–Whitney test and Fisher’s exact test for the continuous and categorical variables, respectively, to compare the two groups. We used the Kruskal-Wallis test with Bonferroni correction for the continuous variables to compare among the multiple groups. We used GraphPad Prism 8.4.3 (GraphPad Software, San Diego, CA) for all analyses, with significance set at *P* < 0.05.

## Results

During the study period, there were 17 285 deliveries across the participating institutions, of which 397 had severe PPH excluding placental abruption and 232 had placental abruptions, with 159 and 56 enrolled, respectively. Cases lacking the required data were excluded, and 171 (131 severe PPH and 40 placental abruption) were ultimately used for the study. Table 1 outlines their characteristics. Maternal age was 35 (22–45) years with 44% primiparas and 19% multifetal pregnancies. Cesarean section was performed in 59% of cases. No maternal deaths occurred during this study.

**Table 1:** Maternal and neonatal characteristics for each category and all participants

Maternal characteristics	WBLH (*n* = 9)	WBLL (*n* = 12)	CFC (*n* = 62)	NCP (*n* = 88)	All participants (*N* = 171)
Age (years)	36 (27–40)	32 (26–39)	33.5 (22–45)	36 (24–45)	35 (22–45)
Primipara	1 (11.1)	5 (41.7)	26 (41.9)	43 (48.9)	75 (43.9)
Body mass index at delivery (kg/m^2^)	23.9 (20.6–31)	23.2 (19.1–28.1)	26 (20.2–38.6)	24.9 (17.8–37.2)	25 (17.8–38.6)
Gestational age at delivery (weeks)	38 (27–39)	38.5 (31–41)	37 (22–41)	38 (23–41)	37 (22–41)
HDP complicated	2 (22.2)	2 (16.7)	21 (33.9)	16 (18.2)	41 (24)
Mode of delivery					
Cesarean section	5 (55.6)	5 (41.7)	47 (75.8)	44 (50)	101 (59.1)
Instrumental vaginal	1 (11.1)	2 (16.7)	2 (3.2)	14 (15.9)	19 (11.1)
Multifetal pregnancy	3 (33.3)	1 (8.3)	15 (24.2)	13 (14.8)	32 (18.7)
Neonatal characteristics					
Body weight at delivery (g)	2814 (977–3772)	2764 (1835–3732)	2557 (510–4074)	2950 (600–3902)	2758 (510–4074)
Apgar score at 1 min	8 (1–8)	8 (2–10)	8 (0–10)	8 (0–10)	8 (0–10)
Umbilical arterial pH	7.29 (7.11–7.31)	7.28 (6.81–7.42)	7.28 (6.56–7.39)	7.28 (6.61–7.44)	7.28 (6.56–7.44)

Data presented median (range) or no. (%). *HDP* Hypertension disorder of pregnancy. WBLH, WBLL, CFC, and NCP indicate a condition with whole blood loss with high bleeding rate, whole blood loss with low bleeding rate, coagulation factor consumption, and non-coagulopathy, respectively

**Fig. 1:**
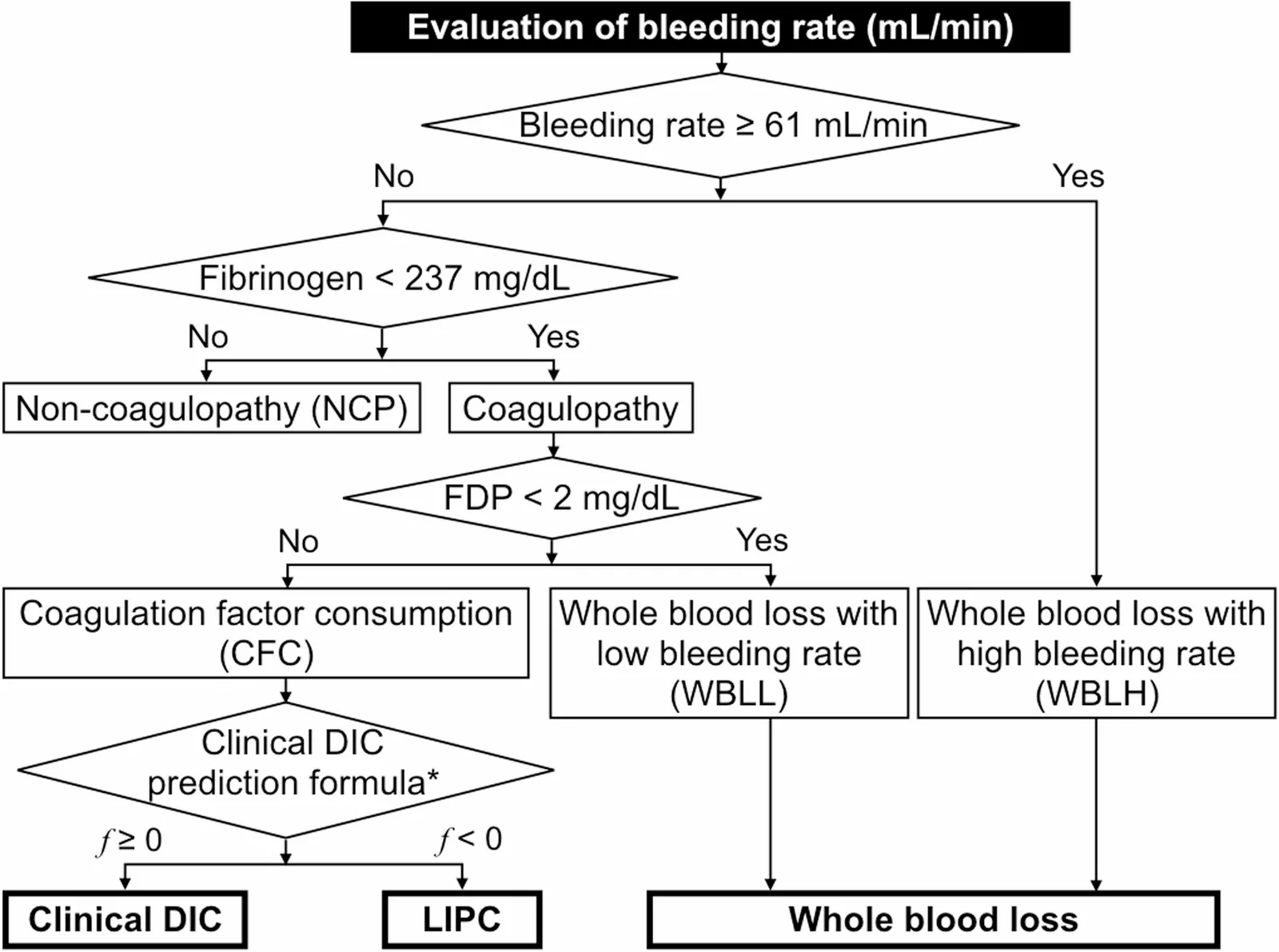
Flowchart of algorithm for classifying underlying pathologies using bleeding rate, fibrinogen, and FDP. Bleeding rate is defined as blood loss at initial blood sampling (mL) divided by time of initial blood sampling after delivery (min). * Clinical DIC prediction formula: *f (FDP*,* fibrinogen) = FDP–fibrinogen/3–60 (mg/dL)*; *f* ≥ 0, DIC; *f* < 0, LIPC [7]. DIC, disseminated intravascular coagulation; FDP, fibrin/fibrinogen degradation products; LIPC, locally initiated and progressive consumptive coagulopathy

Figure 1 shows a flowchart of the algorithm. The 95th percentile value of the 171 participants for bleeding rate was 61 mL/min. These 171 comprised WBLH (*n* = 9), NCP (*n* = 88), WBLL (*n* = 12), and CFC (*n* = 62). Of the CFC cases, 3 had clinical DIC and 59 had LIPC. The underlying pathologies in PPH can ultimately be classified into clinical DIC, LIPC, and whole blood loss. As there were only 3 clinical DIC cases, we compared laboratory and clinical data among four categories – WBLH, WBLL, NCP, and CFC – which comprised clinical DIC and LIPC (Table 2).

**Table 2:** Laboratory and clinical data for each category and all participants

Laboratory data	WBLH (*n* = 9)	WBLL (*n* = 12)	CFC (*n* = 62)	NCP (*n* = 88)	All participants (*N* = 171)
Hemoglobin (g/dL)	8.2 (4.7–11.5)	6.3 (3.5–10)	7.35 (4.5–11.3)	8.95 (5.3–13)	8.3 (3.5–13)
Platelet counts (×1000/µL)	151 (89–302)	132 (70–219)	129.5 (39–273)	188.5 (68–332)	154 (39–332)
Prothrombin time–INR	1.07 (0.92–1.2)	1.15 (0.91–10)	1.06 (0.88–1.84)	0.98 (0.79–1.26)	1.04 (0.79–10)
Fibrinogen (mg/dL)	262 (183–543)	212 (50–231)	168.5 (38–235)	325 (237–678)	244 (38–678)
FDP (mg/dL)	1.19 (0.72–5.39)	0.91 (0.29–1.82)	13.1 (2.1–96)	2.6 (0.38–55)	3.48 (0.29–96)
D-dimer (mg/dL)	0.4 (0.25–1.17)	0.41 (0.09–0.64)	2.56 (0.45–49.1)	0.9 (0.17–7.61)	1.09 (0.09–49.1)
TAT (ng/mL)	10.5 (6.4–30.7) ^a^	10.65 (2.5–26)	42.8 (6.8–120) ^b^	16.5 (2.3–120) ^c^	19.9 (2.3–120) ^d^
PIC (µg/mL)	0.55 (0.4–2.6) ^a^	0.75 (0.3–3.3)	6.65 (0.7–68.3) ^b^	1.8 (0.3–17.2) ^c^	2.6 (0.3–68.3) ^d^
Clinical data					
Time of initial blood sampling after delivery (min)	30 (18–60)	96 (30–354)	120 (34–840)	120 (20–1290)	120 (18–1290)
Amount of blood loss (mL)					
At initial blood sampling	2300 (1100–3775)	2392 (830–3300)	2218 (404–4815)	1978 (290–10000)	2100 (290–10000)
Total	2898 (2020–3975)	2799 (850–4826)	2570 (468–13000)	2347 (290–10000)	2464 (290–13000)
Bleeding rate (mL/min)	74 (61.1–127)	25.8 (2.5–58.7)	16 (1.3–47.9)	16.1 (0.5–60.2)	16.7 (0.5–127)
Shock index	0.88 (0.6–1.17)	1 (0.5–2.8)	0.7 (0.4–2.1)	0.71 (0.4–1.56)	0.75 (0.4–2.8)
Administration volume of clear fluids (mL)					
At initial blood sampling	2000 (500–2500)	1500 (200–3500)	1500 (200–4500)	1500 (200–8000)	1500 (200–8000)
Total	3500 (2000–6000)	3500 (1000–8000)	3500 (200–7000)	3000 (200–8000)	3000 (200–8000)
Transfusion volume of RCC (mL)					
At initial blood sampling	0 (0–0)	0 (0–1400)	0 (0–2240)	0 (0–3080)	0 (0–3080)
Total	0 (0–840)	840 (0–1680)	560 (0–5880)	0 (0–3080)	0 (0–5880)
RCC administration	2 (22.2)	11 (91.7)	37 (59.7)	19 (21.6)	69 (40.4)
Transfusion volume of FFP (mL)					
At initial blood sampling	0 (0–0)	0 (0–0)	0 (0–3600)	0 (0–2400)	0 (0–3600)
Total	0 (0–960)	240 (0–1920)	120 (0–4800)	0 (0–2400)	0 (0–4800)
FFP administration	2 (22.2)	6 (50)	31 (50)	14 (15.9)	53 (31)

Data presented median (range) or no. (%)

*FDP* Fibrin/fibrinogen degradation products, *FFP* Fresh frozen plasma, *INR* International normalized ratio, *PIC* Plasmin-α2–plasmin inhibitor complex, *RCC* Red cell contents, *TAT* Thrombin–antithrombin complex. WBLH, WBLL, CFC, and NCP indicate a condition with whole blood loss with high bleeding rate, whole blood loss with low bleeding rate, coagulation factor consumption, and non-coagulopathy, respectively

^a^ Data are available for 8 patients. ^b^ Data are available for 60 patients. ^c^ Data are available for 81 patients. ^d^ Data are available for 161 patients

Table 3 shows a comparison of the same data in Tables 1 and 2 between clinical DIC and LIPC. The fibrinogen levels for DIC were lower (*P* < 0.001) than for LIPC, and the FDP (*P* < 0.001), TAT (*P* < 0.05), and PIC (*P* < 0.01) levels for DIC were higher than for LIPC. Hemoglobin levels, bleeding rate, time of initial blood sampling after delivery, and shock index did not differ between DIC and LIPC.

**Table 3:** Comparison of maternal and neonatal characteristics, and laboratory and clinical data between clinical DIC and LIPC

Maternal characteristics	Clinical DIC (*n* = 3)	LIPC (*n* = 59)	*P* value
Age (years)	32 (28–36)	34 (22–43)	0.47
Primipara	2 (66.7)	24 (40.7)	0.18
Body mass index at delivery (kg/m^2^)	25.6 (20.6–28.7)	26.1 (20.2–33.8)	0.64
Gestational age at delivery (weeks)	37 (32–38)	37 (22–40)	0.81
Hypertension disorder of pregnancy complicated	2 (66.7)	19 (32.2)	0.26
Mode of delivery			
Cesarean section	3 (100)	44 (74.6)	0.99
Instrumental vaginal	0 (0)	2 (3.4)	0.99
Multifetal pregnancy	1 (33.3)	14 (23.7)	0.99
Neonatal characteristics			
Body weight at delivery (g)	2550 (1326–2661)	2564 (510–3368)	0.49
Apgar score at 1 min	8 (1–8)	8 (0–10)	0.78
Umbilical arterial pH	7.228 (7.225–7.296)	7.29 (6.56–7.39)	0.53
Laboratory data			
Hemoglobin (g/dL)	7.5 (4.5–10.1)	7.3 (4.9–11.3)	0.92
Platelet counts (×1000/µL)	96 (76–144)	131 (39–243)	0.29
Prothrombin time–international normalized ratio	1.53 (1.46–1.84)	1.06 (0.88–1.49)	< 0.001
Fibrinogen (mg/dL)	62 (38–79)	180 (64–235)	< 0.001
Fibrin/fibrinogen degradation products (mg/dL)	96 (94.73–96)	11.11 (2.09–96)	< 0.001
D-dimer (mg/dL)	42.55 (13.54–49.1)	2.43 (0.45–26.69)	< 0.001
TAT (ng/mL)	120 (120–120)	39.3 (6.8–120) ^a^	< 0.05
PIC (µg/mL)	28.4 (17.7–68.3)	6.2 (0.7–43.3) ^a^	< 0.01
Clinical data			
Time of initial blood sampling after delivery (min)	90 (78–240)	120 (34–440)	0.57
Amount of blood loss (mL)			
At initial blood sampling	2215 (1300–2215)	2276 (404–4010)	0.49
Total	2950 (2700–3801)	2450 (468–4110)	0.45
Bleeding rate (mL/min)	24.6 (5.4–28.4)	15.7 (1.3–47.9)	0.64
Shock index	0.7 (0.62–1.24)	0.7 (0.4–1)	0.77
Administration volume of clear fluids (mL)			
At initial blood sampling	1500 (500–2000)	1500 (200–2500)	0.45
Total	4500 (3500–5500)	3000 (200–6000)	0.21
Transfusion volume of red cell contents (mL)			
At initial blood sampling	0 (0–0)	0 (0–560)	0.59
Total	1120 (840–1960)	480 (0–1680)	< 0.05
Red cell contents administration	3 (100)	34 (57.6)	0.27
Transfusion volume of fresh frozen plasma (mL)			
At initial blood sampling	0 (0–0)	0 (0–1680)	0.59
Total	960 (720–1920)	0 (0–1920)	< 0.05
Fresh frozen plasma administration	3 (100)	28 (47.5)	0.24

Data presented as median (range) or no. (%). DIC and LIPC indicate a condition with disseminate intravascular coagulation and locally initiated and progressive consumptive coagulopathy, respectively

*PIC* Plasmin-α2–plasmin inhibitor complex, *TAT* Thrombin–antithrombin complex

^a^ Data are available for 57 patients

**Fig. 2:**
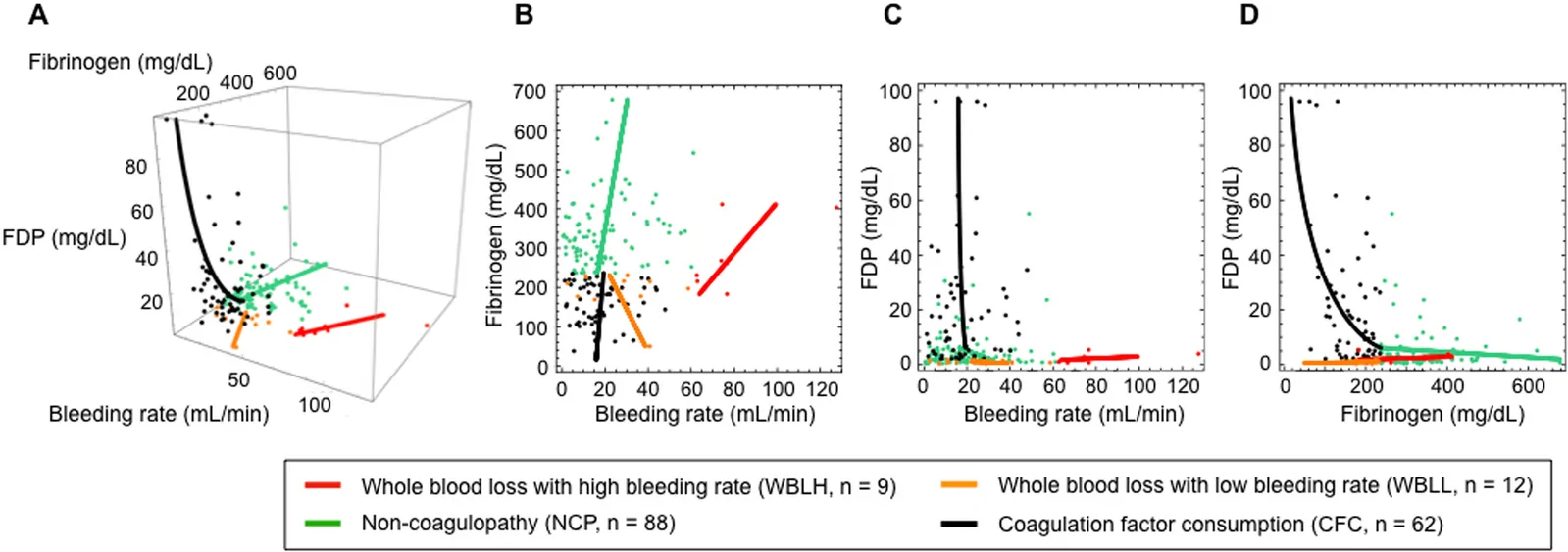
Distributions of cases with each category on three-dimensional space and two-dimensional planes. **A** Space comprising bleeding rate, fibrinogen, and FDP. **B** Plane comprising bleeding rate and fibrinogen. **C** Plane comprising bleeding rate and FDP. **D** Plane comprising fibrinogen and FDP. The NCP and CFC formed a nearly continuous pathological continuum, with a fibrinogen level of 237 mg/dL serving as the boundary (**B**, **D**). Although WBLH and WBLL had different conditions regarding bleeding rate (**C**) and fibrinogen (**B**), they shared a common feature: the absence of elevated FDP (**D**). FDP, fibrin/fibrinogen degradation products

Figure 2 shows the distributions of cases for each category across three-dimensional space and two-dimensional planes representing bleeding rate and fibrinogen, bleeding rate and FDP, and fibrinogen and FDP. The NCP (shown in green) and CFC (black) formed a nearly continuous pathological continuum, with a fibrinogen level of 237 mg/dL serving as the boundary (Fig. 2B and D). Although WBLH (red) and WBLL (orange) had different conditions regarding bleeding rate (Fig. 2C) and fibrinogen (Fig. 2B), they shared a common feature: the absence of elevated FDP (Fig. 2D).

**Fig. 3:**
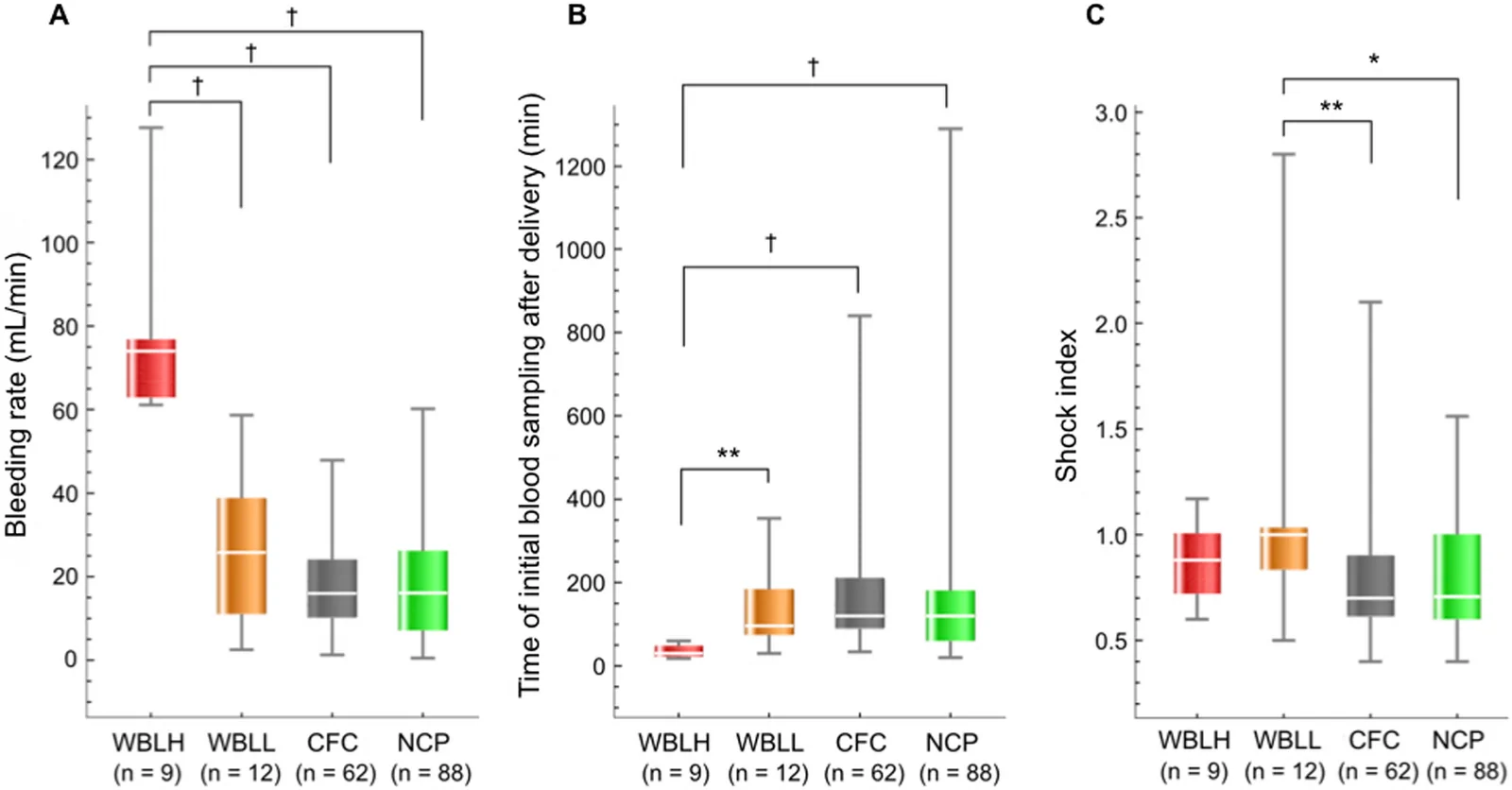
Comparison of clinical features among the four categories. **A** Bleeding rate in WBLH was higher (*P* < 0.0001) than in the other three categories. **B** Times of initial blood sampling after delivery in WBLH were significantly earlier than in the other three categories. **C** Shock indices in WBLL were higher than those in CFC (*P* < 0.01) and NCP (*P* < 0.05). The boxes, middle line, and whiskers represent the 25th and 75th percentiles, median value, and maximum and minimum values, respectively. *, *P* < 0.05; **, *P* < 0.01; †, *P* < 0.0001. WBLH, WBLL, CFC, and NCP indicate a condition with whole blood loss with high bleeding rate, whole blood loss with low bleeding rate, coagulation factor consumption, and non-coagulopathy, respectively

Figure 3 compares the clinical data among the four categories. The *P* values for the multiple comparisons among WBLH, WBLL, CFC, and NCP were < 0.0001, < 0.0001, and < 0.05 for bleeding rate, time of initial blood sampling after delivery, and shock index, respectively. As defined above, the bleeding rate in WBLH was higher (*P* < 0.0001) than that in the other three categories. For WBLL, it tended to be higher than in CFC (*P* = 0.181) and NCP (*P* = 0.196). Notably, time of initial blood sampling after delivery in WBLH was significantly earlier than in the other three categories. The shock index in WBLL was higher than in CFC (*P* < 0.01) and NCP (*P* < 0.05) but did not differ from that in WBLH.

**Fig. 4:**
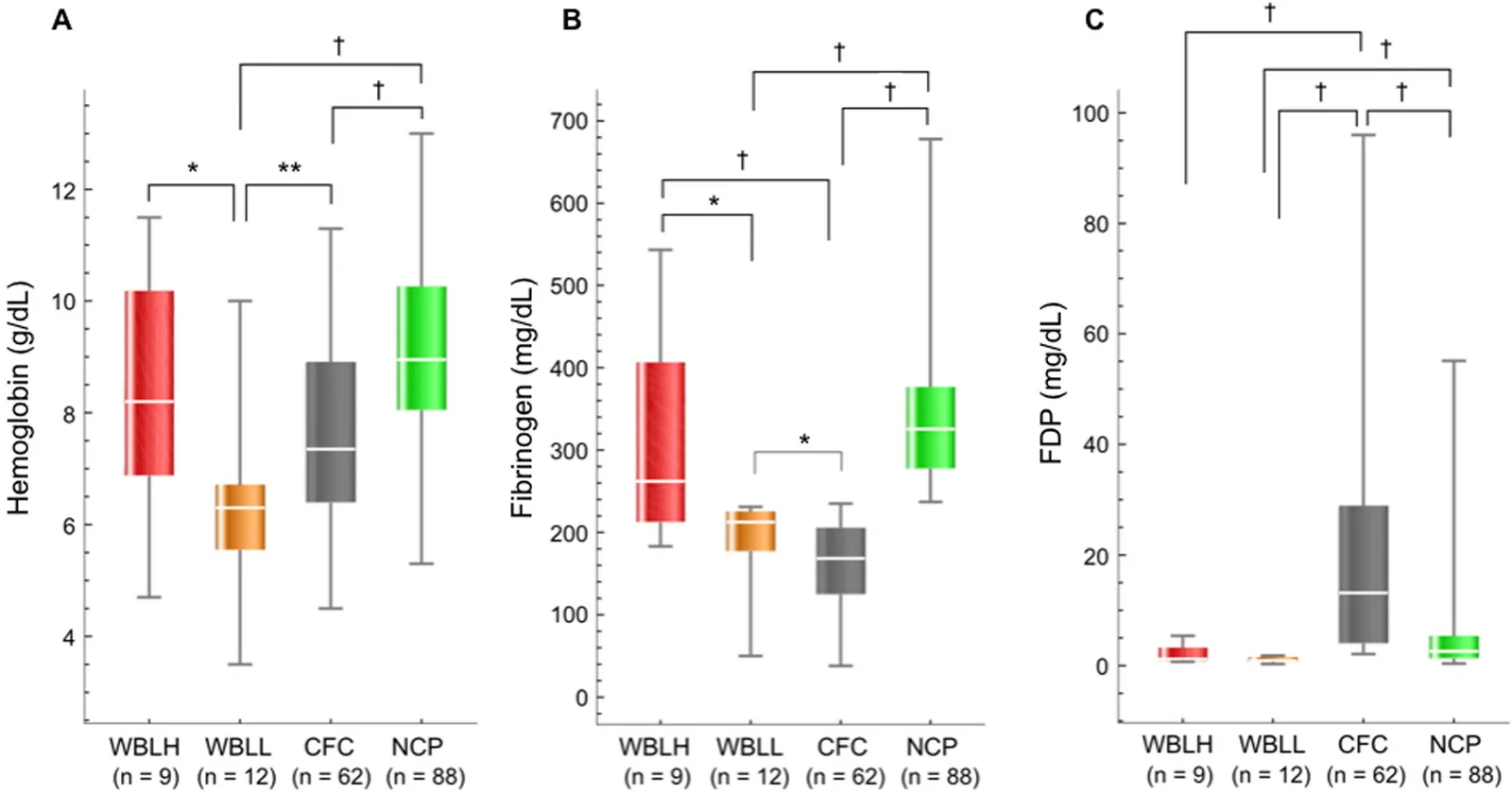
Laboratory data comparison for the four categories. **A** Hemoglobin levels in WBLL were significantly lower than in the other three categories. **B** Fibrinogen levels in CFC were significantly lower than in the other three categories. **C** FDP levels in CFC were higher (*P* < 0.0001) than in the other three categories. FDP levels did not differ between WBLH and WBLL. The boxes, middle line, and whiskers represent the 25th and 75th percentiles, median value, and maximum and minimum values, respectively. *, *P* < 0.05; **, *P* < 0.01; †, *P* < 0.0001. WBLH, WBLL, CFC, and NCP indicate a condition with whole blood loss with high bleeding rate, whole blood loss with low bleeding rate, coagulation factor consumption, and non-coagulopathy, respectively. FDP, fibrin/fibrinogen degradation products

Figure 4 compares the laboratory data among the four categories. The *P* values for the multiple comparisons among WBLH, WBLL, CFC, and NCP were all < 0.0001 for hemoglobin, fibrinogen, and FDP, respectively. The hemoglobin and fibrinogen levels in WBLH did not differ from those in NCP. The hemoglobin levels in WBLL were significantly lower than those in the other three categories. The fibrinogen and FDP levels in CFC were significantly lower and higher, respectively, than those in the other three categories. The FDP levels in WBLH (median, 1.19 mg/dL) did not differ from those in WBLL (median, 0.91 mg/dL).

Table 4 shows the causes of bleeding in each category. Uterine atony was the most common underlying pathology in WBLH (89%; 8/9) and WBLL (58%; 7/12). The underlying pathologies in CFC varied, but there were 19 cases of placental abruption other than NCP, of which 18 (95%) were in CFC. Two cases of hemolysis, elevated liver enzymes, and low platelets (HELLP) syndrome were in CFC.

**Table 4:** Causes of bleeding for each category and all participants

Causes of bleeding	WBLH (*n* = 9)	WBLL (*n* = 12)	CFC (*n* = 62)		NCP (*n* = 88)	All participants (*N* = 171)
			Clinical DIC (*n* = 3)	LIPC (*n* = 59)		
Uterine atony	8 (88.9)	7 (58.3)	1 (33.3)	10 (16.9)	29 (33)	55 (32.2)
Placental abruption	0 (0)	1 (8.3)	0 (0)	18 (30.5)	21 (23.9)	40 (23.4)
Surgical or birth canal trauma	1 (11.1)	2 (16.7)	2 (66.7)	19 (32.2)	18 (20.5)	42 (24.6)
Adherent or retained placenta	0 (0)	2 (16.7)	0 (0)	4 (6.8)	12 (13.6)	18 (10.5)
Placenta previa	0 (0)	0 (0)	0 (0)	5 (8.5)	8 (9.1)	13 (7.6)
HELLP syndrome	0 (0)	0 (0)	0 (0)	2 (3.4)	0 (0)	2 (1.2)
Uterine inversion	0 (0)	0 (0)	0 (0)	1 (1.7)	0 (0)	1 (0.6)

Data presented as no. (%). *HELLP* Hemolysis, elevated liver enzymes and low platelets. WBLH, WBLL, CFC, DIC, LIPC, and NCP indicate a condition with whole blood loss with high bleeding rate, whole blood loss with low bleeding rate, coagulation factor consumption, disseminated intravascular coagulation, locally initiated and progressive consumptive coagulopathy, and non-coagulopathy, respectively

## Discussion

In this study, we demonstrated the development of an algorithm that classifies underlying pathologies in PPH into four categories – WBLH, WBLL, NCP and CFC – using 61 mL/min bleeding rate, 237 mg/dL fibrinogen, and 2 mg/dL FDP. CFC was then further classified into clinical DIC and LIPC using the formula shown in Fig. 1. For PPH management, it has been reported that fibrinogen < 200 mg/dL is a predictive marker of worsening bleeding [17, 18, 19] and that fibrinogen and FDP are essential parameters for diagnosing DIC [7, 20, 21]. Additionally, several reports have indicated a need to further consider the parameter of bleeding rate [22, 23]. However, to our knowledge, no reports have quantitatively and comprehensively classified the underlying pathologies in PPH by these clinical parameters.

The initial diagnosis of PPH in a clinical setting is usually based on clinical impression, and bleeding rate is a key factor in the decision-making process in PPH management [22, 24]. In this study, bleeding rate was the algorithm’s first parameter; cases with bleeding rate ≥ 61 mL/min were defined as WBLH. This condition had two distinctive features. First, the initial blood sampling time after delivery (median, 30 min) was markedly earlier than for the other three categories (Fig. 3), indicating that the attending physicians responded promptly to the high-flow hemorrhaging in these cases. Consequently, the hemoglobin and fibrinogen levels in the WBLH group did not differ from NCP without coagulopathy (Fig. 4). Second, the FDP levels in WBLH were low and not differ significantly from those in WBLL with FDP < 2 mg/dL (Fig. 4), suggesting that effective hemostatic thrombi had not formed. Additionally, analysis of the administration volume of clear fluids did not indicate massive fluid administration in the WBLH group. Consequently, we can reasonably conclude that the underlying pathologies in WBLH were characterized by whole blood loss rather than dilutional coagulopathy [25].

WBLL was characterized by a significant decrease in hemoglobin (median, 6.3 g/dL), mild decrease in fibrinogen (median, 212 mg/dL), and elevated shock index (median, 1.0) (Figs. 3 and 4). Shock index is considered a clinical indicator of hypovolemic shock [26]. A shock index > 0.9 in PPH patients can suggest severe PPH [27]. These findings support the hypothesis that WBLL is characterized by whole blood loss. The bleeding rate in WBLL (median, 25.8 mL/min) tended to be higher than in NCP and CFC, but lower than in WBLH (median, 74 mL/min) (Table 2; Fig. 3A), which may have delayed medical intervention and deteriorated clinical findings. To our knowledge, this is the first quantitative demonstration that coagulopathy without elevated FDP (< 2 mg/dL) may represent a clinical emergency.

CFC was characterized by a moderate decrease in hemoglobin (median, 7.35 g/dL) and significant decrease in fibrinogen (median, 168.5 mg/dL) (Fig. 4). These findings are consistent with those reported in patients with placental abruption, a primary etiology of consumptive coagulopathy [28, 29]. Additionally, the shock index in CFC (median, 0.7) showed no deterioration (Fig. 3C), which also is consistent with the results reported above [28]. The main pathology of DIC involves abnormal activation of the coagulation-fibrinolytic systems. Elevated TAT and PIC – molecular markers reflecting activation of the coagulation and fibrinolysis systems, respectively – are essential for DIC diagnosis [13]. TAT and PIC in cases of clinical DIC might be significantly higher than those in LIPC (Table 3), thus, fulfilling the diagnostic requirements. However, this study cannot draw definitive conclusions due to the very small number of DIC subgroups (only 3 cases). We should cautiously interpret the comparison between DIC and LIPC.

The statistical analysis suggests that underlying pathologies in each category differ from those observed in a normal state, which demonstrate separation between categories but do not independently validate the underlying pathologies. The algorithm using universal markers, measurable at many facilities, may be clinically useful in managing PPH. For example, if fibrinogen decreases < 237 mg/dL, deterioration of the patient’s condition due to coagulopathy should be monitored. If no FDP increase (< 2 mg/dL) is observed, we might then consider that DIC is unlikely regardless of the bleeding state, and continued bleeding with non-elevated FDP is a warning of progression to hemorrhagic shock. The suggested treatments for each category as hypotheses for future evaluation could be as follows: uterotonic administration in WBLH; for WBLL, anti-shock therapy including blood products transfusion and surgical hemostasis in trauma case; for CFC, correction of hypofibrinogenemia with fresh frozen plasma or fibrinogen concentrates [19], especially in DIC cases.

In examining the causes of bleeding, uterine atony notably accounted for 89% of WBLH and 58% of WBLL (Table 4). Uterine atony occurs after childbirth when uterine contractions ineffectively compress the spiral arteries providing blood to the placenta [30]. Therefore, we can assume that acute arterial bleeding would prevent the formation of effective hemostatic thrombi in WBLH and WBLL. Yamaguchi et al. [31] reported that a PPH case resistant to treatment showing arterial contrast extravasation on dynamic computed tomography (PRACE) was associated with the interventional procedure. Although FDP levels were not measured in that report, some cases of PRACE caused by arterial bleeding may have an underlying pathology without FDP elevation. The causes of bleeding in CFC were diverse; however, all cases of HELLP syndrome (*n* = 2) and 95% (18/19) of cases of placental abruption with coagulopathy were categorized under this condition, making these relationships biologically consistent, as HELLP syndrome and placental abruption were typical causes of consumptive coagulopathy [2, 4].

### Limitations

This study did have several limitations. The enrollment rates were low – at 33% (131 of 397 cases) for PPH and 17% (40 of 232 cases) for placental abruption – and it is preferable in case series to report consecutive patients to avoid selection bias in enrollment [32]. Bleeding rate measurement may be difficult in practice because the amount of blood loss can be often inaccurate, and the timing of initial blood sampling depends on clinical judgment. The intervention timing and the methods used to treat bleeding also varied among facilities and were not standardized, which possibly influenced the laboratory or clinical data. For practical applicability of the algorithm, we should consider that rapid FDP testing might not yet be routinely available in all institutions. Additionally, as no maternal deaths occurred in this study, we were unable to examine the algorithm’s validity histopathologically, especially in clinical DIC. Further confirmation via additional data is needed, which is common among all studies on PPH diagnosis and/or management. Finally, in CFC, we were unable to examine the degree of involvement of blood loss. Certain cases with mildly elevated FDP may be primarily attributed to blood loss rather than coagulation factor consumption.

## Conclusions

This multicenter case series demonstrated that underlying pathologies in severe PPH and placental abruption could be quantitatively characterized using three clinical parameters: bleeding rate, fibrinogen, and FDP. The laboratory and clinical data for each underlying pathology classified using our algorithm developed in this study reflected each of the pathophysiological characteristics, thereby supporting the biological plausibility of the algorithm, but requiring external prospective validation before clinical implementation. This study can serve as a foundation for future research on the pathophysiology in PPH.

## Data Availability

The datasets generated during this study are available from the corresponding author upon reasonable request.

## Abbreviations

FDP: Fibrin/fibrinogen degradation products
FFP: Fresh frozen plasma
HELLP: Hemolysis, elevated liver enzymes and low platelets
HDP: Hypertension disorder of pregnancy
INR: International normalized ratio
NHO: National Hospital Organization
PIC: Plasmin-α2–plasmin inhibitor complex
PPH: Postpartum hemorrhage
PRACE: PPH that is resistant to treatment showing arterial contrast extravasation on dynamic computed tomography
RCC: Red cell contents
TAT: Thrombin–antithrombin complex
WBLH: Whole blood loss with high bleeding rate
WBLL: Whole blood loss with low bleeding rate
NCP: Non-coagulopathy
CFC: Coagulation factor consumption
DIC: Disseminated intravascular coagulation
LIPC: Locally initiated and progressive consumptive coagulopathy
